# Increasing Incidence of Mucormycosis in University Hospital, Belgium

**DOI:** 10.3201/eid1609.100276

**Published:** 2010-09

**Authors:** Veroniek Saegeman, Johan Maertens, Wouter Meersseman, Isabel Spriet, Eric Verbeken, Katrien Lagrou

**Affiliations:** Author affiliation: Leuven University Hospitals, Leuven, Belgium

**Keywords:** Fungi, invasive fungal disease, incidence, voriconazole, hematologic neoplasms, hematopoietic stem cell transplantation, mucormycosis, opportunistic infections, Belgium, dispatch

## Abstract

To determine why incidence of mucormycosis infections was increasing in a large university hospital in Belgium, we examined case data from 2000–2009. We found the increase was not related to voriconazole use but most probably to an increase in high-risk patients, particularly those with underlying hematologic malignancies.

In September 2009, Bitar et al. reported an increasing incidence of mucormycosis in France from 1997 through 2006 (*1*). Their epidemiologic study was based on International Classification of Diseases, 10th Revision (ICD-10), codes extracted from hospital information systems from an estimated 95% of public and private hospitals in France. This study is particularly interesting because population-based estimates of the incidence of mucormycosis are scarce (*2*). However, as the authors state, ICD code–based evaluations have limitations; such limitations are mainly related to difficulties encountered with the diagnosis of mucormycosis in clinical practice. Conventional diagnostic tools lack sensitivity. Moreover, distinction between colonization and infection is problematic in the absence of invasive procedures or autopsy data. Thus, epidemiologic studies on mucormycosis are often hampered by the limited number of documented cases.

Several surveys have been conducted in large US transplant centers. Based on these studies, the increasing incidence of mucormycosis has been linked to the widespread use of voriconazole prophylaxis in high-risk patients (*3**,**4*). In contrast with the study in France, these surveys have the limitation of focusing on particular risk groups, mainly cancer patients, and do not provide a general estimate on the incidence of mucormycosis. In our study, we determined the incidence rate of invasive mucormycosis in a large university hospital in Belgium over a 10-year period and reviewed the clinical data of the patients with proven or probable disease.

## The Study

Case finding was based on culture and pathology data and not on ICD codes. Culture data from all patients hospitalized at the University Hospitals of Leuven (Belgium) from 2000 through 2009 were retrieved from the Sirscan 2000 system (i2a, Montpelier, France). Computer queries were run to retrieve all reports from histologic examinations of tissue specimens to which the Leiden code “fungus” had been assigned (*5*). Medical records of all patients with culture or histopathologic evidence for *Mucorales* spp. organisms were reviewed, and all patients were classified as having proven or probable invasive mucormycosis according to European Organization for Research and Treatment of Cancer and Mycoses Study Group (EORTC-MSG) criteria (*6*).

**Figure 1:**
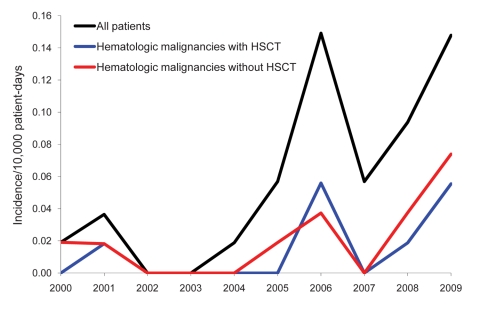
Incidence of mucormycosis cases in a hospital in Belgium, 2000–2009. HSCT, hematopoietic stem cell transplantation.

Over this 10-year time period, 31 patients with mucormycosis were identified: 21 with proven and 10 with probable disease. The annual incidence increased from 0.019 cases/10,000 patient-days in 2000 to 0.148 cases/10,000 patient-days in 2009 (Spearman correlation coefficient 0.75; p = 0.01) (Figure 1). The incidence increased mainly from 2005–2009. Averaged over the 10 years, the incidence was 0.058/10,000 patient-days. The sex ratio (M/F) was 16/15; mean age was 54 years (median 59 years, range 12–79 years). Nineteen (61%) of 31 mucormycosis patients had a hematologic disorder; 8 patients underwent a hematopoietic stem cell transplantation. Other underlying conditions were diabetes mellitus (n = 2), solid organ transplantation (n = 4), surgery/trauma (n = 4), autoimmune disorder (n = 1), and HIV infection (n = 1).

**Figure 2:**
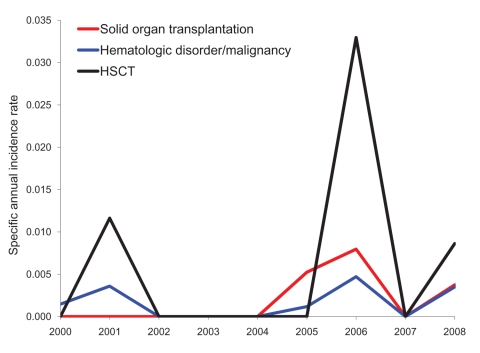
Specific annual incidence rate for the risk groups solid organ transplantation, hematologic disorder/malignancy, and hematopoietic stem cell transplantation (HSCT) in a hospital in Belgium, 2000–2008.

The number of patients belonging to the risk groups diabetes mellitus, solid organ transplantation, hematologic disorder/malignancy, and hematopoietic stem cell transplantation was determined based on International Classification of Diseases, 9th Revision, Clinical Modification (ICD-9-CM), codes entered in the hospital information system (available up to the end of 2008). An increase in the number of diabetes mellitus (p<0.01) and hematologic disorder/malignancy (p<0.01) patients (Spearman correlation; Table) was seen from 2000 through 2008. The specific annual incidence rate (defined as the number of mucormycosis cases per risk group over the number of risk patients per year) for these 4 risk groups did not increase significantly during this period (Spearman correlation; p>0.01) (Table; Figure 2).

**Table:** Specific annual incidence rate of mucormycosis cases per risk group in a hospital in Belgium, 2000–2008*

Year	Diabetes mellitus	Solid organ transplantation	Hematologic disorder/ malignancy	HSCT
2000	0/3,087	0/235	1/684	0/96
2001	0/3,328	0/217	2/557	1/86
2002	0/3,319	0/207	0/650	0/87
2003	0/3,662	0/245	0/775	0/118
2004	1/3,845	0/217	0/787	0/126
2005	1/4,121	1/191	1/853	0/103
2006	0/4,235	2/251	5/1,061	3/91
2007	0/4,328	0/279	0/942	0/121
2008	0/4,477	1/267	3/859	1/116
p value for the increase in				
No. patients per risk group	<0.01	0.12	<0.01	0.19
Specific annual incidence rate	0.81	0.11	0.91	0.55

*Values are no. cases per risk group/no. patients admitted per year with that risk factor, unless otherwise indicated. Data for risk group patients based on codes from the International Classification of Diseases, 9th Revision, Clinical Modification, available 2000–2008. HSCT, hematopoietic stem cell transplantation.

None of the 31 patients had received voriconazole prophylaxis (per institutional policy). Mucormycosis developed in 1 patient while that patient was receiving voriconazole therapy for invasive aspergillosis.

Clinical signs included pulmonary (51.5%), disseminated (23%), cutaneous (13%), sinus (6.5%), cerebral (3%), and mycetoma (3%) mucormycosis. A high percentage (45%) of co-infections with *Aspergillus* spp. was recorded. Five of the 6 patients showing a halo sign on chest computed tomography scan were co-infected with *Aspergillus* spp.

The death rate was 65%. For 48% of the patients, death was directly related to mucormycosis infection.

## Conclusions

We report an increasing incidence rate of invasive mucormycosis not related to the use of voriconazole, either prophylactically or therapeutically, in a university hospital in Belgium. Pongas et al. calculated that 49% of mucormycosis cases were encountered in the setting of voriconazole prophylaxis (*3*). A case–control observational study identified voriconazole prophylaxis as an independent risk factor for the development of mucormycosis (*7*). However, in our series, none of the 31 patients had received voriconazole prophylaxis, and mucormycosis developed in only 1 patient who was receiving voriconazole therapy for invasive aspergillosis.

Compared with the study from France (6.8%), our study reported a notably high percentage (23%) of cases of disseminated disease (*1*). However, high incidences, up to 17% of cases, were also reported from other recent clinical registries (*8**,**9*). In our hospital, the number of patients with disseminated mucormycosis infection may be increasing; 1 case was diagnosed in 2000, 1 in 2005, 2 in 2008, and 3 in 2009. This finding possibly reflects the growing population of severely immunosuppressed patients.

The death rate of 65% is consistent with death rates reported in recent clinical registries (≈50%) but appears to be much higher than rates reported in the study in France. However, this discrepancy might be merely a reflection of differences in case definitions (*1**,**8**,**9*). We expect death rates to be higher in studies or surveillances that apply more stringent case definitions; cases with only fungal colonization, not invasive disease, are less likely to be included in these studies.

During the 10-year period, the most important risk group, namely patients with a hematologic disorder/malignancy, expanded considerably. The population of patients with diabetes also increased, but mucormycosis in this host population is quite rare in our institution (only 2 cases in 10 years). A decreasing number of published mucormycosis cases since the 1990s in patients with diabetes mellitus as underlying illness was reported in a large review of the literature (*10*). This finding could be a consequence of better glycemic control and decreasing rates of diabetic ketoacidosis and because of the widespread use of statins in patients with diabetes (*11*).

Several limitations of our study can be mentioned. First, 5 cases of mucormycosis were diagnosed on the basis of histopathologic findings only. Second, the numbers of the risk populations were derived from ICD-9-CM codes and are thus subject to all inaccuracies associated with discharge coding of patients. Third, we used the EORTC-MSG criteria for classification of cases, but, unlike other filamentous fungal pathogens that target immunocompromised hosts, *Mucorales* spp. organisms infect a broader and more heterogeneous population. Thus, invasive mucormycosis also occurs in immunocompetent patients without any host factor as defined by these criteria. As such, invasive procedures are necessary in immunocompetent patients to enable the diagnosis of proven invasive mucormycosis according to the EORTC-MSG criteria. However, we estimate the effect of this limitation to be minor because in immunocompetent patients biopsy procedures are generally feasible, particularly because mucormycosis is most often seen as a cutaneous infection in these patients. Finally, our results are single-institution data and are not necessarily representative of other institutions or other geographic regions.

In conclusion, the increasing incidence of mucormycosis shown in our study was not related to the prior use of voriconazole. Rather, the increasing incidence was most probably associated with an increasing number of patients who had an underlying hematologic malignancy.
